# Identification of new biomarkers for Acute Respiratory Distress Syndrome by expression-based genome-wide association study

**DOI:** 10.1186/s12890-015-0088-x

**Published:** 2015-08-19

**Authors:** Dmitry N. Grigoryev, Dilyara I. Cheranova, Suman Chaudhary, Daniel P. Heruth, Li Qin Zhang, Shui Q. Ye

**Affiliations:** Laboratory of Translational Studies and Personalized Medicine, Moscow Institute of Physics and Technology, Dolgoprudny, Moscow Region, Russian Federation; Division of Experimental and Translational Genetics, Department of Pediatrics, Children’s Mercy Hospitals and Clinics, Kansas City, MO USA; Department of Biomedical and Health Informatics, University of Missouri Kansas City School of Medicine, Kansas City, MO USA

**Keywords:** Expression-based genome-wide association studies, Acute respiratory distress syndrome, Gene expression, Biomarkers, Microarray, Genomics

## Abstract

**Background:**

Accumulated to-date gene microarray data on Acute Respiratory Distress Syndrome (ARDS) in the Gene Expression Omnibus (GEO) represent a rich source for identifying new unsuspected targets and mechanisms of ARDS. The recently developed expression-based genome-wide association study (eGWAS) for analysis of GEO data was successfully used for analysis of gene expression of comparatively noncomplex adipose tissue, 75 % of which is represented by adipocytes. Although lung tissue is more heterogenic and does not possess a prevalent cell type for driving gene expression patterns, we hypothesized that eGWAS of ARDS samples will generate biologically meaningful results.

**Methods:**

The eGWAS was conducted according to (Proc Natl Acad Sci U S A 109:7049-7054, 2012) and genes were ranked according to *p* values of chi-square test.

**Results:**

The search of GEO retrieved 487 ARDS related entries. These entries were filtered for multiple qualitative and quantitative conditions and 219 samples were selected: mouse *n*_sham/ARDS_ = 67/92, rat *n* = 13/13, human cells *n* = 11/11, canine *n* = 6/6 with the following ARDS model distributions: mechanical ventilation (MV)/cyclic stretch *n* = 11; endotoxin (LPS) treatment *n* = 8; MV + LPS *n* = 3; distant organ injury induced ARDS *n* = 3; chemically induced ARDS *n* = 2; *Staphylococcus aureus* induced ARDS *n* = 2; and one experiment each for radiation and shock induced ARDS. The eGWAS of this dataset identified 42 significant (Bonferroni threshold *P* < 1.55 × 10^−6^) genes. 66.6 % of these genes, were associated previously with lung injury and include the well known ARDS genes such as IL1R2 (*P* = 4.42 × 10^−19^), IL1β (*P* = 3.38 × 10^−17^), PAI1 (*P* = 9.59 × 10^−14^), IL6 (*P* = 3.57 × 10^−12^), SOCS3 (*P* = 1.05 × 10^−10^), and THBS1 (*P* = 2.01 × 10^−9^). The remaining genes were new ARDS candidates. Expression of the most prominently upregulated genes, CLEC4E (*P* = 4.46 × 10^−14^) and CD300LF (*P* = 2.31 × 10^−16^), was confirmed by real time PCR. The former was also validated by in silico pathway analysis and the latter by Western blot analysis.

**Conclusions:**

Our first in the field application of eGWAS in ARDS and utilization of more than 120 publicly available microarray samples of ARDS not only justified applicability of eGWAS to complex lung tissue, but also discovered 14 new candidate genes which associated with ARDS. Detailed studies of these new candidates might lead to identification of unsuspected evolutionarily conserved mechanisms triggered by ARDS.

**Electronic supplementary material:**

The online version of this article (doi:10.1186/s12890-015-0088-x) contains supplementary material, which is available to authorized users.

## Background

Acute Respiratory Distress Syndrome (ARDS) is a devastating illness associated with systemic inflammatory response to infection or severe injury [1]. Sepsis, trauma, shock, surgery and other causes of systemic inflammation can lead to ARDS. The clinical presentation of ARDS is characterized by profuse pulmonary edema, acute lung inflammation with recruitment of neutrophils and disruption of the alveolar-capillary barrier. Several studies by our group replicated these characteristics of ARDS in both cell culture (cyclic stretch) and rodent models (mechanical ventilation, endotoxin (LPS) treatment, and ARDS induced by kidney ischemia reperfusion injury) [2, 3]. The role of inflammation in ARDS has typically been studied in relation to either infection and sepsis or trauma and visceral organ injury. Although progress has been made in the understanding of ARDS etiologies, there is pausity of studies that focus on common responses of the lung to both septic and aseptic challenges.

While in the last decade, a large amount of data from different models of ARDS was collected and became available to the scientific community, the systematic analysis of this remarkable data substrate was not actively conducted. Therefore, we intended to analyze this big data using newly developed expression-based genome-wide association studies (eGWAS), which was tested successfully on adipose tissue [4]. However, the majority of cells in adipose tissue is represented by adipocytes (75 %), with the other 25 % shared between vasculature and connective tissue [5]. The lung alveolar tissue on the other hand does not have a prevalent cell type, which might drive the transcriptional response to an injury, and is comprised of type I epithelial cells (8 %), alveolar macrophages (9 %), type II epithelial cells (16 %), capillary endothelial cells (30 %), and cells of the interstitial space (37 %) [6].

Therefore, we hypothesized that despite variable contribution to gene expression by different alveolar cell types, eGWAS will be efficient for the analysis of the transcriptional response by lung tissue to an injury. To test this hypothesis we collected and simultaneously analyzed more than 120 publicly available microarray samples of ARDS.

## Methods

### Data

The ARDS datasets were collected from Gene Expression Omnibus (GEO, NCBI, (http://www.ncbi.nlm.nih.gov/geo) public functional genomics data repository. The GEO database was searched for “acute lung injury,” which returned 60 hits, and “lung injury,” which returned 487 hits (as of September 2014). Most of these entries were individual samples that duplicated samples, which were already combined into existing data sets. The elimination of these duplicates reduced the obtained data to 48 sets (Additional file 1: Table S1). These sets were further filtered down to 31 data sets using the following criteria: 1) the array samples must represent genome wide studies by the established microarray platforms with the number of interrogated sequences >5000, thus excluding custom platforms designed for a specific organ or pathway, which introduces tissue or biological process bias; 2) the experimental settings must contain untreated (either placebo or sham) and unmodified (wild type) subjects; 3) biological replicate n ≥ 3 per each sham or experimental group; 4) proper signal distribution as identified by the significance of microarray (SAM) plot.

Authors believe that all data uploaded from GEO involving human subjects have been obtained with the approval of appropriate ethics committees by Silva et al. and Eltzschig et al. (Additional file 1: Table S1).

### Expression-based genome-wide association study (eGWAS)

The eGWAS was conducted as described previously [4]. Briefly, to estimate differences between groups of samples from ARDS subjects and sham controls, raw postquantitation microarray data were reanalyzed by SAM 2.0 software. Then the *P* values from the number of positive/negative experiments for each gene and sum of the number of positive/negative experiments for all other genes were calculated using a 2 × 2 chi-square or a Fisher’s exact test. The probe IDs across different microarray platforms for mouse, rat, and human were linked using the array information library universal navigator (AILUN) tool (http://ailun.stanford.edu). The probes that remained unmatched by the AILUN tool were linked to the AILUN created mouse-rat-human dataset via their gene symbol entries.

Given that canine and human samples were represented by only 1 and 2 experiments, respectively the species-related weighting algorithm [7] was not applicable.

### Pathway analysis

The pathway analysis, which identifies the most relevant biological processes to a specific list of candidate genes, was conducted using the Ingenuity Pathways Knowledge Base tool (IPA, Ingenuity Systems, Inc., Redwood City, CA.) as described previously [3].

### Automated literature search

PubMatrix (multiplex literature mining tool) analysis was conducted as described previously [7]. We restricted our search to human symbols approved by HUGO Gene Nomenclature Committee (HGNC), which were enriched by all aliases and former (discontinued) symbols for selected candidate genes (http://www.genenames.org).

### Cecal ligation and puncture model

All mouse experiments were approved by the University of Missouri Kansas City Institutional Animal Care and User Committee. To confirm the unversal nature of the response to the ARDS stimuli by our candidates, we employed the mouse septic model - cecal ligation and puncture (CLP), which was not present in the GEO data collection (Additional file 1: Table S1). We selected the most commonly used time point (24 h after injury) for the CLP septic model [8]. Animals (*n* = 6) were anesthetized and laparotomy performed. The cecum was exposed and ligated. In three mice, the cecum was punctured twice using 16G needle and then squeezed to extrude contents in a 2 mm of fecal amount. Mice (*n* = 3) without the puncture were used as controls (shams). Wounds were closed. After 24 h mice were sacrificed and the lungs were collected for histological, real-time RT-PCR, and Western blot studies. All mouse experiments were approved by the University of Missouri Kansas City Institutional Animal Care and User Committee.

### Histological studies and Real-time RT-PCR

For histological studies, lungs were perfused first with PBS followed by 4 % paraformaldehyde, and then embedded in paraffin. 5 μm thick slices were prepared and stained with H&E according to standard protocol.

The real time PCR of mRNAs extracted from lung tissue was conducted as previously described [7]. Slight modifications were made according to the manufacturer’s new protocol. Briefly, the 384-well microtiter plate setting of a ViiA™ 7 Real-Time PCR System (Applied Biosystems) was employed. TaqMan® Predeveloped Assay Reagent mouse β-actin (REF 435234E, probe dye VIC-MGB) was used as an internal control for normalization. TaqMan® Gene Expression assay for mouse *Clec4e* and *Cd300lf* were purchased from Applied Biosystems Inc. (Mm 01183703_m1 and Mm 00467508_m1, respectively). All experimental protocols were based on manufactures’ recommendation using the TaqMan® Universal Master Mix II (P/N 4440039). A relative quantitative method was used to calculate corresponding transcript levels relative to actin expression. The significance of the obtained difference in signal between sham and CLP mice was assessed using t-tests where *p* < 0.05 was considered significant.

### Western blotting

Immediately after euthanizing the mice, the lungs were perfused with PBS prior to tissue proteins extraction with lysis buffer. Protein concentration was determined by BCA assay (Thermo Scientific, prod # 23227). Equal total proteins (20 μg) for each lung cell lysate sample were analyzed by SDS-PAGE followed by Western blotting on PVDF membrane. Anti-CD300LF (Proteintech Group, Inc, cat# 13334-1-AP) antibody was used. Monoclonal anti-actin antibody (Santa Cruz, cat. # SC-47778) was used as a loading control. Protein signal was revealed by FluorChem M chemiluminescence (Proteinsimple™).

## Results and discussion

The search of the GEO repository returned submissions related to lung injury from the past 8 years with a total of 487 samples of *in vivo* and *in vitro* models of ARDS, which covered the most common biological experimental models: rodent, canine, and cultures of human cells. The filtering for inclusion criteria outlined in Materials and Methods retained a total of 97 control and 122 ARDS samples: mouse *n*_sham/ARDS_ = 67/92, rat *n* = 13/13, human cells *n* = 11/11, canine *n* = 6/6. All these samples were derived from 31 *in vivo* and *in vitro* experimental settings (ES) that were grouped into 8 common models: 1) mechanical ventilation (MV)/cyclic stretch ES = 11; 2) LPS treatment ES = 8; 3) MV + LPS ES = 3; 4) distant organ injury induced ARDS ES = 3; 5) chemically induced ARDS ES = 2; 6) *Staphylococcus aureus* induced ARDS ES = 2; and one experiment for each 7) radiation and 8) shock induced ARDS (Additional file 1: Table S1).

**Fig. 1:**
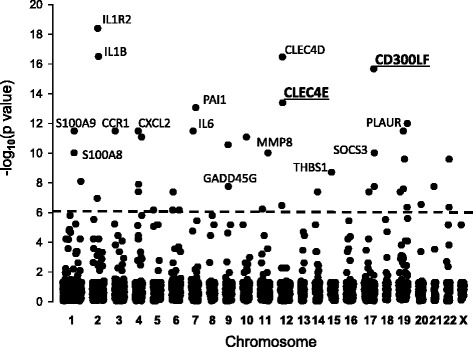
eGWAS of combined 8 ARDS models. The plot depicts chi-square *P* values (−log_10_) on the y axis arranged by chromosomal location (x axis). *P* values for each gene were calculated for 97 sham-operated or vehicle exposed control microarray samples and 122 ARDS microarray samples as the likelihood of finding repeated differential expression compared with expected using *χ* ^*2*^ analysis. Out of 32160 tested gene entries, 42 genes demonstrated a significant differential expression. The dashed line indicates the Bonferroni threshold (*P* = 1.55 × 10^−6^). The gene symbols indicate known ARDS genes that are most significantly associated with ARDS throughout all tested models, where underscored symbols indicate new candidate genes

Cross-referencing of these microarrays resulted in a dataset of 32160 gene entries. eGWAS analysis ranked these genes first by the likelihood that repeated differential expression for a given gene was due to chance, then controlled for multiple-hypothesis testing using Bonferroni threshold (*P* < 1.55 × 10^−6^), which identified 42 candidate genes (Fig. 1, Additional file 2: Table S2).

To identify relevance of our candidates to ARDS, we linked 42 genes to 3 terms: “lung”, “lung injury”, and “acute lung injury” using the PubMatrix tool. This approach identified 23 candidates (54.7 %) as previously linked to ARDS genes (at least one citation with the term “acute lung injury”), which justifies the suitability of eGWAS for the analysis of lung injury (Table 1). The actual Score(d) and fold changes for these genes in each model can be found in Additional file 3: Table S3.

**Table 1:** PubMatrix analysis

Symbol	Lung	Lung injury	Acute lung injury
IL1R2	11	3	1
IL1B	59	11	4
CLEC4D	1	1	0
CD300LF	0	0	0
CLEC4E	1	0	0
PAI1	548	159	91
LILRB4	4	0	0
S100A9	88	8	2
CCR1	84	10	6
CXCL2	600	250	134
IL6	332	55	24
PLAUR	146	16	6
CCRN4L	0	0	0
CH25H	3	0	0
NFIL3	2	1	1
S100A8	96	12	6
MMP8	20	5	4
SOCS3	82	15	8
BCL3	2	1	1
MAFF	3	0	0
THBS1	140	12	1
RHOU	3	0	0
CXCL3	13	3	2
GADD45G	10	0	0
SPHK1	32	6	0
SAMSN1	3	0	0
ZFP36	11	0	0
CXCL1	458	163	91
CDKN1A	714	35	5
ARG2	23	1	0
CCL3	305	54	26
JUNB	55	3	0
PLA2G7	2	0	0
ARID5A	0	0	0
SLPI	184	28	10
APOLD1	1	0	0
CSF2RB	18	1	0
FPR2	42	6	4
ADM	440	21	8
OSMR	7	0	0
TREM1	39	21	3
TNFAIP3	16	5	3

This approach also identified 5 novel genes (at least one citation with the term “lung injury”) and 14 new genes (0 links to “lung injury”). Surprisingly, among the top five genes identified in our study to be associated with ARDS, only *IL1R2* and *IL1B* were known ARDS genes [3], the other three genes were a novel C-type lectin domain family 4, member D *CLEC4D* gene [9] and new genes CD300 molecule-like family member F (*CD300LF*) [10] and C-type lectin domain family 4, member E (*CLEC4E*) [11] (Fig. 1 and Table 1).

**Fig. 2:**
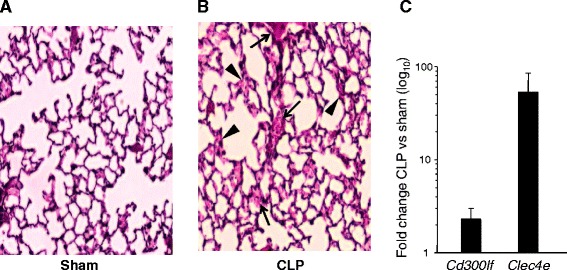
Histopathology of CLP affected lungs and expression pattern of novel ARDS candidate genes. *Panels* ***a***-***b*** *:* Light micrographs of H&E stained lung sections from sham (**a**) and CLP (**b**) mice. The lung histopathology in CLP-challenged mice demonstrates broadening of alveolar septa with sparse monocyte infiltration (arrowheads) and hemorrhage in septa (arrow). Original magnification, 200×. *Panel* **c** *:* The expression of *Clec4e* and *Cd300lf* genes in whole mouse lung is represented by horizontal bars. The error bars are standard deviations among three samples. The real time PCR was conducted using commercially available TaqMan^TM^ reactions

To confirm the commonality of expressional changes of our candidates to the ARDS stimuli we utilized a CLP septic model, which was not present among the models obtained from GEO (Additional file 1: Table S1). The histological study of this additional model confirmed the acute injury to lung tissue (Fig. 2a and 2b). Real-time PCR analysis of *Cd300lf* and *Clec4e* expression in the CLP model demonstrated upregulation of both genes (Fig. 2c), similar to that detected by eGWAS.

**Fig. 3:**
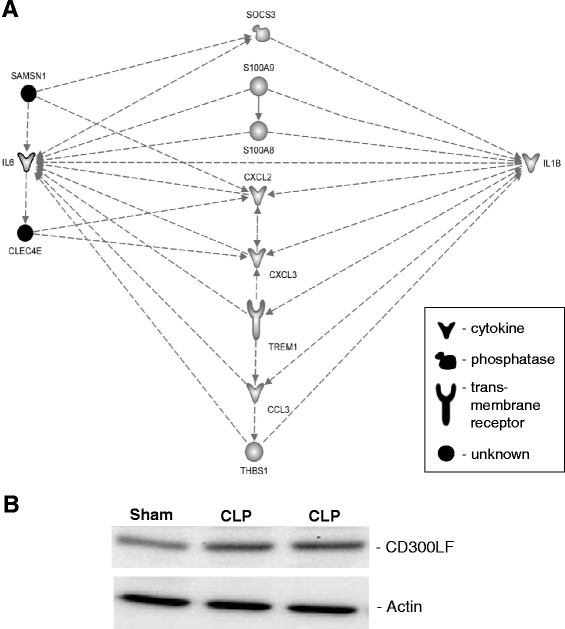
In silico and in vitro validation of *Clec4e* and *Cd300lf* gene candidates. *Panel* **a**: The top gene network generated by IPA for 42 ARDS gene candidates. This network was reduced to genes that posses at least three established connections to members of the network. Relationships between genes are represented by solid lines (binding), solid arrows (direct activation), or broken arrows (indirect activation). Arrows point to the element on which an action is performed. Black molecules represent new candidate genes. Grey molecules represent significant ARDS genes. *Panel* **b**: The Western blot analysis of Cd300lf protein expression. Each lane contains 20 μg of lung tissue lysate. The top bands represent signals of Cd300lf protein in the sham and two CLP challenged mice. The bottom bands represent signals of actin, which was used as a loading control

Interactive network analysis of 42 candidates incorporated one of our candidates, *CLEC4E,* into the network of the well-known ARDS genes (Fig. 3a), thus indirectly validating involvement of this gene in the pathogenesis of ARDS. Indeed, this gene can be a potential biomarker for ARDS. *CLEC4E* is expressed in activated macrophages and codes for a cell-surface receptor, which recognizes a wide variety of ligands including damaged cells, fungi and mycobacteria [12]. Moreover, there is a recent report on the protective role of *CLEC4E* in sepsis caused by *K. pneumonia* [13], moreover, *CLEC4E* binds to another lung specific microorganism *Streptococcus pneumoniae* [14]. These reports indirectly link our new candidate to the lung pathology and warrant the new studies of this gene in the ARDS settings.

Our next candidate, *CD300LF*, codes for an inhibitory receptor of the Ig superfamily of myeloid cells. To confirm the significance of *CD300LF* expression, we measured its protein expression by Western blot analysis, which confirmed upregulation of our candidate (Fig. 3b). There are no reports on the potential link of *CD300LF* to a lung pathology, however it has been shown that upregulation of this gene has a protective role in acute brain injury [15], therefore it will be enticing to investigate whether upregulation of this candidate will play a similar protective role in acute lung injury.

## Conclusions

In the present study, we investigated molecular signatures of 8 ARDS models in four different biological systems (mouse, rat, dog, and human) using eGWAS. eGWAS identified 42 ARDS candidate genes, two-thirds of which was previously linked to lung injury, which justifies the eGWAS utility for investigating differential gene expression in complex tissues.

Our first in the field exploration of ARDS using eGWAS of 120 microarray samples of lung injury identified 14 new molecular targets common to different species and models, suggesting new players in the evolutionarily conserved mechanisms triggered during ARDS. These findings will guide further research in the field of molecular targeting of ARDS and will open new unsuspected avenues for translational research. We expect that further studies of our newly discovered candidates will lead to the development of new biomarkers for ARDS.

## Additional files

Additional file 1:
**Table S1.** Detailed representation of data obtained from GEO. ARDS gene expression submissions were retrieved from GEO using two terms “Acute lung injury” and “Lung injury”, which resulted in 23 and 25 data sets, respectively. These 48 entries were filtered down to 31 entries according to conditions described in Methods. The reason for filtering out an experiment is provided. (XLSX 16 kb) Additional file 2:
**Table S2.** Chi-square values of cross-referenced genes. Given that different microarray platforms have multiple probes for a single gene, the cross-referencing of such platforms generates numerous combinations of expression values for a given gene (32160 in this study). Chi-square value was calculated for each of these combinations and combination with the best chi-square value for a given gene was retained, which resulted in 3152 unique gene entries. These genes were linked to human genome and plotted against their location (Fig. 1). (XLS 185 kb) Additional file 3:
**Table S3.** Contribution of each ARDS model to the overall gene expression signal of an ARDS gene candidate. Each dataset obtained from GEO was reanalyzed using SAM 2.0. The d-score and fold change values for 42 gene candidates were extracted from SAM 2.0 outputs and reported according to the ARDS model. (XLSX 45 kb)

## Abbreviations

ARDS: Acute respiratory distress syndrome
CLP: Cecal ligation and puncture
eGWAS: Expression-based genome-wide association study
GEO: Gene expression omnibus
HGNC: HUGO gene nomenclature committee
IPA: Ingenuity pathway analysis
IRI: Ischemia reperfusion injury
LPS: Lipopolysaccharide
MV: Mechanical ventilation
SAM: Significance analysis of microarray
